# The chromosomal genome sequence of the sponge, *Rhopaloeides odorabile* Thompson, Murphy, Bergquist & Evans, 1987 (Dictyoceratida: Spongiidae) and its associated microbial metagenome sequences

**DOI:** 10.12688/wellcomeopenres.26110.1

**Published:** 2026-04-10

**Authors:** Nicole S. Webster, Sara C. Bell, Heidi M. Luter, Dirk Erpenbeck, Ute Hentschel, Graeme Oatley, Elizabeth Sinclair, Eerik Aunin, Noah Gettle, Camilla Santos, Michael Paulini, Haoyu Niu, Victoria McKenna, Rebecca O’Brien, Wellcome Sanger Institute Tree of Life Management, Samples and Laboratory team, Wellcome Sanger Institute Scientific Operations: Sequencing Operations, Wellcome Sanger Institute Tree of Life Core Informatics team, EBI Aquatic Symbiosis Genomics Data Portal Team, Aquatic Symbiosis Genomics Project Leadership

**Affiliations:** 1 Institute for Marine and Antarctic Studies, University of Tasmania, Hobart, Tasmania, Australia; 2 Australian Centre for Ecogenomics, University of Queensland, St Lucia, Queensland, Australia; 3 Australian Institute of Marine Science, Townsville, Queensland, Australia; 4 Department of Earth and Environmental Sciences, Palaeontology & Geobiology, LMU München, Munich, Germany; 5 GEOMAR Helmholtz Centre for Ocean Research Kiel, Kiel, Germany; 6 Tree of Life Programme, Wellcome Sanger Institute, Hinxton, England, UK

**Keywords:** Rhopaloeides odorabile; sponge; genome sequence; chromosomal; Dictyoceratida; microbial metagenome assembly

## Abstract

We present a genome assembly from an individual *Rhopaloeides odorabile* (Porifera; Demospongiae; Dictyoceratida; Spongiidae). The genome sequence has a total length of 291.63 megabases. Most of the assembly (98.17%) is scaffolded into 17 chromosomal pseudomolecules. The mitochondrial genome has also been assembled, with a length of 16.42 kilobases. From the metagenome data, we recovered 162 bins, of which 96 were high-quality MAGs. *R. odorabile* displays a characteristic high microbial abundance sponge profile, with MAGs representing diverse phyla (i.e., Acidobacteriota, Pseudomonadota, and Chloroflexota) and candidate phyla (i.e., *Ca.* Latescibacteria, *Ca.* Poribacteria, and *Ca.* Tectomicrobia).

## Species taxonomy

Eukaryota; Opisthokonta; Metazoa; Porifera; Demospongiae; Keratosa; Dictyoceratida; Spongiidae; *Rhopaloeides*; *Rhopaloeides odorabile* Thompson, Murphy, Bergquist & Evans, 1987 (NCBI:txid476028)

## Background

*Rhopaloeides odorabile* is a viviparous Dictyoceratid sponge (Demospongiiae), common on the Great Barrier Reef, which dribble spawns tufted parenchymella larvae over a period of 5–6 weeks during the Austral summer ( Whalan *et al.*, 2007). *R. odorabile* possesses an unusual group of bioactively important C20 diterpenes which show variation in yield with changing environmental parameters, such as depth and light exposure ( Thompson *et al.*, 1987). The habitat distribution of *R. odorabile* correlates with light availability, although it does not host photosymbiotic microorganisms ( Bannister *et al.*, 2011).

*R. odorabile* is an intensively studied high-microbial-abundance (HMA) sponge. The cultivated bacterial community is dominated by an Alphaproteobacterium that is highly sensitive to environmental stress ( Webster *et al.*, 2001b; Webster & Hill, 2001), whereas the uncultivated microbial community in *R. odorabile* includes an enormous diversity of bacteria ( Webster *et al.*, 2001c, 2010), archaea ( Webster *et al.*, 2001a) and viruses ( Laffy *et al.*, 2016). Although the symbionts have been genomically characterised ( Robbins *et al.*, 2021), the roles of these symbionts in this sponge remain enigmatic, although their high specificity and stability over temporal, geographic and environmental gradients imply a strong link to sponge health. To maintain the sponge microbiome, *R. odorabile* employs dual evolutionary modes of vertical transmission ( Webster *et al.*, 2010) and acquisition of symbionts from the rare seawater biosphere ( Tout *et al.*, 2017; Webster *et al.*, 2010).

In terms of environmental sensitivity, adult *R. odorabile* and their microbial symbionts have a strict thermal threshold of 32°C ( Bennett *et al.*, 2016; Pantile & Webster, 2011; Simister *et al.*, 2012; Webster *et al.*, 2008). At this temperature, *R. odorabile* alters its filtration and feeding activity ( Massaro *et al.*, 2012), there is disruption to nutritional interdependence and molecular interactions between host and symbionts ( Bennett *et al.*, 2016; Fan *et al.*, 2013) and a concomitant increase in endogenous retro-transcribing viruses within the Caulimorviridae and Retroviridae families ( Laffy *et al.*, 2019). In contrast, larval *R. odorabile* exhibit a markedly higher thermal tolerance, with no adverse health effects detected at temperatures below 36°C ( Webster *et al.*, 2011). *R. odorabile* larvae can also survive exposure to high levels of nutrients ( Simister *et al.*, 2012), as well as high concentrations of petroleum hydrocarbons; however, their ability to settle and metamorphose is adversely affected at environmentally relevant oil concentrations ( Luter *et al.*, 2019).

Whilst there have been no reports of widespread disease in *R. odorabile*, a spongin-boring bacterium, *Pseudoalteromonas agarivorans* ( Choudhury *et al.*, 2014; Choudhury *et al.*, 2015), which produces a potent collagenolytic enzyme ( Mukherjee *et al.*, 2009) has been identified as a primary agent of disease ( Webster *et al.*, 2002).

**Figure 1.**
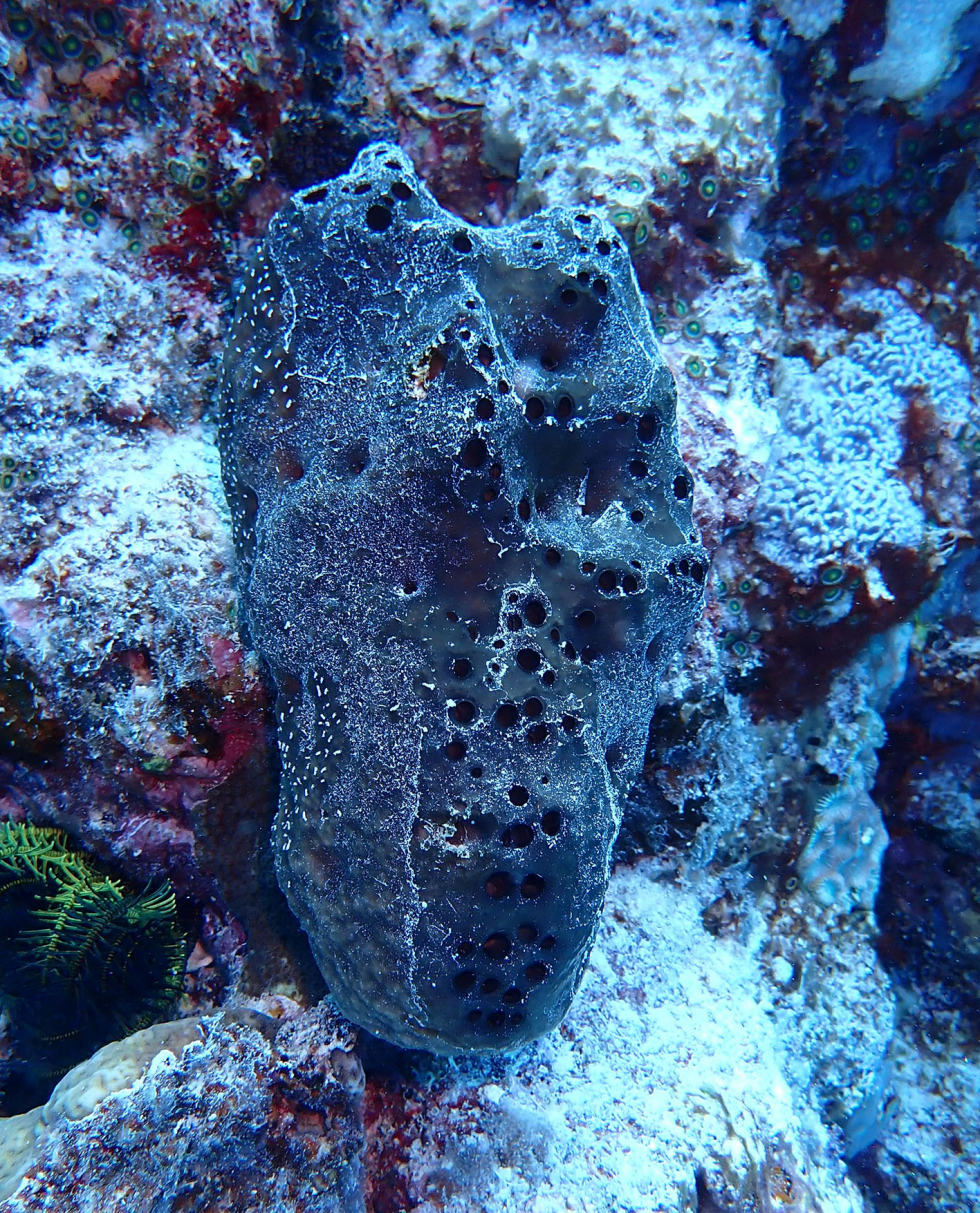
*In situ* image of the *Rhopaloeides odorabile* individual (odRhoOdor1.1) used for genome sequencing. The specimen was collected from Davies Reef on the Great Barrier Reef by SCUBA diving (underwater photography by Heidi Luter).

We present a chromosome-level genome sequence for *R. odorabile.* The assembly was produced using the Tree of Life pipeline from a specimen collected from Davies Reef, Great Barrier Reef, Queensland, Australia (  Figure 1). This assembly was generated as part of the Aquatic Symbiosis Genomics project.

## Methods

### Sample acquisition

The specimen used for genome sequencing was an adult *Rhopaloeides odorabile* (specimen ID GHC0000117, ToLID odRhoOdor1; Figure 1), collected from Davies Reef, Great Barrier Reef, Queensland, Australia (latitude 18.915, longitude 147.7036) on 2020-09-23 by SCUBA diving. The specimen was transported in a shaded aquaria to the National Sea Simulator at the Australian Institute of Marine Science (Townsville, Australia) where it was snap frozen in liquid nitrogen and stored at -80°C until further processing. The specimen was collected and identified by Nicole Webster (AIMS). The same specimen was used for RNA sequencing.

### Nucleic acid extraction

Protocols for high molecular weight (HMW) DNA extraction developed at the Wellcome Sanger Institute (WSI) Tree of Life Core Laboratory are available on protocols.io ( Howard *et al.*, 2025). The odRhoOdor1 sample was weighed and triaged to determine the appropriate extraction protocol. Tissue was disrupted using the sponge squeezing protocol. HMW DNA was extracted using the Manual MagAttract protocol. We used centrifuge-mediated fragmentation to produce DNA fragments in the 8–10 kb range, following the Covaris g-TUBE protocol for ultra-low input (ULI). Sheared DNA was purified by automated SPRI (solid-phase reversible immobilisation). The concentration of the sheared and purified DNA was assessed using a Nanodrop spectrophotometer and Qubit Fluorometer using the Qubit dsDNA High Sensitivity Assay kit. Fragment size distribution was evaluated by running the sample on the FemtoPulse system.

RNA was extracted from tissue of odRhoOdor1 in the Tree of Life Laboratory at the WSI using the RNA Extraction: Automated MagMax™ *mir*Vana protocol. The RNA concentration was assessed using a Nanodrop spectrophotometer and a Qubit Fluorometer using the Qubit RNA Broad-Range Assay kit. Analysis of the integrity of the RNA was done using the Agilent RNA 6000 Pico Kit and Eukaryotic Total RNA assay.

### PacBio HiFi library preparation and sequencing

Library preparation and sequencing were performed at the WSI Scientific Operations core. Prior to library preparation, the DNA was fragmented to ~10 kb. Ultra-low-input (ULI) libraries were prepared using the PacBio SMRTbell® Express Template Prep Kit 2.0 and gDNA Sample Amplification Kit. Samples were normalised to 20 ng DNA. Single-strand overhang removal, DNA damage repair, and end-repair/A-tailing were performed according to the manufacturer’s instructions, followed by adapter ligation. A 0.85× pre-PCR clean-up was carried out with Promega ProNex beads.

The DNA was evenly divided into two aliquots for dual PCR (reactions A and B), both following the manufacturer’s protocol. A 0.85× post-PCR clean-up was performed with ProNex beads. DNA concentration was measured using a Qubit Fluorometer v4.0 (Thermo Fisher Scientific) with the Qubit HS Assay Kit, and fragment size was assessed on an Agilent Femto Pulse Automated Pulsed Field CE Instrument (Agilent Technologies) using the gDNA 55 kb BAC analysis kit. PCR reactions A and B were then pooled, ensuring a total mass of ≥500 ng in 47.4 μl.

The pooled sample underwent another round of DNA damage repair, end-repair/A-tailing, and hairpin adapter ligation. A 1× clean-up was performed with ProNex beads, followed by DNA quantification using the Qubit and fragment size analysis using the Agilent Femto Pulse. Size selection was performed on the Sage Sciences PippinHT system, with target fragment size determined by Femto Pulse analysis (typically 4–9 kb). Size-selected libraries were cleaned with 1.0× ProNex beads and normalised to 2 nM before sequencing.

The sample was sequenced on a Revio instrument (Pacific Biosciences). The prepared library was normalised to 2 nM, and 15 μL was used for making complexes. Primers were annealed and polymerases bound to generate circularised complexes, following the manufacturer’s instructions. Complexes were purified using 1.2X SMRTbell beads, then diluted to the Revio loading concentration (200–300 pM) and spiked with a Revio sequencing internal control. The sample was sequenced on a Revio 25M SMRT cell. The SMRT Link software (Pacific Biosciences), a web-based workflow manager, was used to configure and monitor the run and to carry out primary and secondary data analysis.

### Hi-C 

***Sample preparation and crosslinking***

 The Hi-C sample was prepared from 20–50 mg of frozen tissue from the odRhoOdor1 sample using the Arima-HiC v2 kit (Arima Genomics). Following the manufacturer’s instructions, tissue was fixed and DNA crosslinked using TC buffer to a final formaldehyde concentration of 2%. The tissue was homogenised using the Diagnocine Power Masher-II. Crosslinked DNA was digested with a restriction enzyme master mix, biotinylated, and ligated. Clean-up was performed with SPRISelect beads before library preparation. DNA concentration was measured with the Qubit Fluorometer (Thermo Fisher Scientific) and Qubit HS Assay Kit. The biotinylation percentage was estimated using the Arima-HiC v2 QC beads.

***Hi-C library preparation and sequencing***

Biotinylated DNA constructs were fragmented using a Covaris E220 sonicator and size selected to 400–600 bp using SPRISelect beads. DNA was enriched with Arima-HiC v2 kit Enrichment beads. End repair, A-tailing, and adapter ligation were carried out with the NEBNext Ultra II DNA Library Prep Kit (New England Biolabs), following a modified protocol where library preparation occurs while DNA remains bound to the Enrichment beads. Library amplification was performed using KAPA HiFi HotStart mix and a custom Unique Dual Index (UDI) barcode set (Integrated DNA Technologies). Depending on sample concentration and biotinylation percentage determined at the crosslinking stage, libraries were amplified with 10–16 PCR cycles. Post-PCR clean-up was performed with SPRISelect beads. Libraries were quantified using the AccuClear Ultra High Sensitivity dsDNA Standards Assay Kit (Biotium) and a FLUOstar Omega plate reader (BMG Labtech).

Prior to sequencing, libraries were normalised to 10 ng/μL. Normalised libraries were quantified again to create equimolar and/or weighted 2.8 nM pools. Pool concentrations were checked using the Agilent 4200 TapeStation (Agilent) with High Sensitivity D500 reagents before sequencing. Sequencing was performed using paired-end 150 bp reads on the Illumina NovaSeq 6000.

### RNA library preparation and sequencing

Libraries were prepared using the NEBNext® Ultra™ II Directional RNA Library Prep Kit for Illumina (New England Biolabs), following the manufacturer’s instructions. Poly(A) mRNA in the total RNA solution was isolated using oligo (dT) beads, converted to cDNA, and uniquely indexed; 14 PCR cycles were performed. Libraries were size-selected to produce fragments between 100–300 bp. Libraries were quantified, normalised, pooled to a final concentration of 2.8 nM, and diluted to 150 pM for loading. Sequencing was carried out on the Illumina NovaSeq X, generating paired-end reads.

### Genome assembly

Prior to assembly of the PacBio HiFi reads, a database of *k*-mer counts ( *k* = 31) was generated from the filtered reads using FastK. GenomeScope2 ( Ranallo-Benavidez *et al.*, 2020) was used to analyse the *k*-mer frequency distributions, providing estimates of genome size, heterozygosity, and repeat content.

The HiFi reads were assembled using Hifiasm ( Cheng *et al.*, 2021) with the --primary option. Haplotypic duplications were identified and removed using purge_dups ( Guan *et al.*, 2020). The Hi-C reads ( Rao *et al.*, 2014) were mapped to the primary contigs using bwa-mem2 ( Vasimuddin *et al.*, 2019), and the contigs were scaffolded in YaHS ( Zhou *et al.*, 2023) with the --break option for handling potential misassemblies. The scaffolded assemblies were evaluated using Gfastats ( Formenti *et al.*, 2022), BUSCO ( Manni *et al.*, 2021) and MERQURY.FK ( Rhie *et al.*, 2020).

The mitochondrial genome was assembled using MitoHiFi ( Uliano-Silva *et al.*, 2023).

### Assembly curation

The assembly was decontaminated using the Assembly Screen for Cobionts and Contaminants ( ASCC) pipeline. TreeVal was used to generate the flat files and maps for use in curation. Manual curation was conducted primarily in PretextView and HiGlass ( Kerpedjiev *et al.*, 2018). Scaffolds were visually inspected and corrected as described by Howe *et al*. (2021). Manual corrections included 495 breaks, 289 joins, and removal of 472 haplotypic duplications. This reduced the scaffold count by 68.2%, reduced the scaffold N50 by 33.4%, and reduced the total assembly length by 30.1%. The curation process is described at https://gitlab.com/wtsi-grit/rapid-curation . PretextSnapshot was used to generate a Hi-C contact map of the final assembly.

### Assembly quality assessment

The Merqury.FK tool ( Rhie *et al.*, 2020) was run in a Singularity container ( Kurtzer *et al.*, 2017) to evaluate *k*-mer completeness and assembly quality for the primary and alternate haplotypes using the *k*-mer database ( *k* = 31) computed prior to genome assembly. The analysis outputs included assembly QV scores and completeness statistics.

The genome was analysed using the BlobToolKit pipeline, a Nextflow implementation of the earlier Snakemake version ( Challis *et al.*, 2020). The pipeline aligns PacBio reads using minimap2 ( Li, 2018) and SAMtools ( Danecek *et al.*, 2021) to generate coverage tracks. It runs BUSCO ( Manni *et al.*, 2021) using lineages identified from the NCBI Taxonomy ( Schoch *et al.*, 2020). For the three domain-level lineages, BUSCO genes are aligned to the UniProt Reference Proteomes database ( Bateman *et al.*, 2023) using DIAMOND blastp ( Buchfink *et al.*, 2021). The genome is divided into chunks based on the density of BUSCO genes from the closest taxonomic lineage, and each chunk is aligned to the UniProt Reference Proteomes database with DIAMOND blastx. Sequences without hits are chunked using seqtk and aligned to the NT database with blastn ( Altschul *et al.*, 1990). The BlobToolKit suite consolidates all outputs into a blobdir for visualisation. The BlobToolKit pipeline was developed using nf-core tooling ( Ewels *et al.*, 2020) and MultiQC ( Ewels *et al.*, 2016), with containerisation through Docker ( Merkel, 2014) and Singularity ( Kurtzer *et al.*, 2017).

## Metagenome assembly

 The metagenome assembly was generated using MetaMDBG ( Benoit *et al.*, 2024). The resulting bin sets of each binning algorithm were optimised and refined using MAGScoT ( Rühlemann *et al.*, 2022). PROKKA ( Seemann, 2014) was used to identify tRNAs and rRNAs in each bin, CheckM ( Parks *et al.*, 2015) (checkM_DB release 2015-01-16) was used to assess bin completeness/contamination, and GTDB-Tk ( Chaumeil *et al.*, 2022) (GTDB release 214) was used to taxonomically classify bins. Taxonomic replicate bins were identified using dRep ( Olm *et al.*, 2017) with default settings (95% ANI threshold). All bins were assessed for quality and categorised as metagenome-assembled genomes (MAGs) if they met the following criteria: contamination ≤ 5%, presence of 5S, 16S, and 23S rRNA genes, at least 18 unique tRNAs, and either ≥ 90% completeness or ≥ 50% completeness with fully circularised chromosomes ( Bowers *et al.*, 2017). Bins that did not meet these thresholds, or were identified as taxonomic replicates of MAGs, were retained as ‘binned metagenomes’ provided they had ≥ 50% completeness and ≤ 10% contamination. A taxonomic tree of the bins was constructed from NCBI classifications using ete3 ( Huerta-Cepas *et al.*, 2016) and visualised with matplotlib.

## Genome sequence report

### Sequence data

PacBio sequencing of the *Rhopaloeides odorabile* specimen generated 141.11 Gb (gigabases) from 15.75 million reads, which were used to assemble the genome. Based on estimated genome size, the sequencing data provided approximately 92× coverage. Hi-C sequencing produced 84.86 Gb from 561.99 million reads, which were used to scaffold the assembly. RNA sequencing data were also generated and are available in public sequence repositories.  Table 1 summarises the specimen and sequencing details.

**Table 1.** Specimen and sequencing data for BioProject PRJEB75563.

Platform	PacBio HiFi	Hi-C	RNA-seq
**ToLID**	odRhoOdor1	odRhoOdor1	odRhoOdor1
**Specimen ID**	GHC0000117	GHC0000117	GHC0000117
**BioSample (source individual)**	SAMEA9614643	SAMEA9614643	SAMEA9614643
**BioSample (tissue)**	SAMEA9614719	SAMEA9614722	SAMEA9614726
**Instrument**	Revio	Illumina NovaSeq 6000	Illumina NovaSeq X
**Run accessions**	ERR13071489; ERR13071486; ERR13071487; ERR13071488	ERR13093662	ERR13669979
**Read count total**	15.75 million	561.99 million	104.26 million
**Base count total**	141.11 Gb	84.86 Gb	15.74 Gb

### Assembly statistics

The primary haplotype was assembled, and contigs corresponding to an alternate haplotype were also deposited in INSDC databases. The final assembly has a total length of 291.63 Mb in 374 scaffolds, with 1 544 gaps, and a scaffold N50 of 16.41 Mb (  Table 2).

**Table 2.** Genome assembly statistics.

**Assembly name**	odRhoOdor1.1
**Assembly accession**	GCA_964237215.1
**Alternate haplotype accession**	GCA_964258675.1
**Assembly level**	chromosome
**Span (Mb)**	291.63
**Number of chromosomes**	17
**Number of contigs**	1 918
**Contig N50**	0.28 Mb
**Number of scaffolds**	374
**Scaffold N50**	16.41 Mb
**Organelles**	Mitochondrion: 16.42 kb

**Figure 2.**
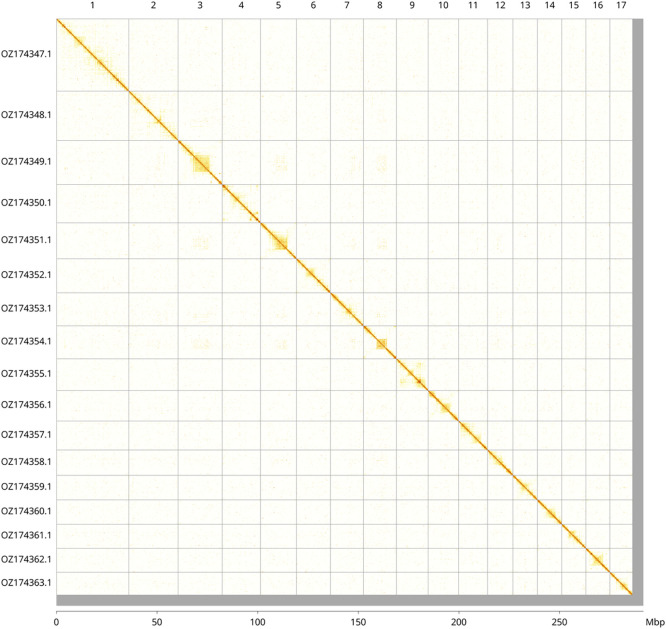
Hi-C contact map of the *Rhopaloeides odorabile* genome assembly. Assembled chromosomes are shown in order of size and labelled along the axes, with a megabase scale shown below. The plot was generated using PretextSnapshot.

Most of the assembly sequence (98.17%) was assigned to 17 chromosomal-level scaffolds. These chromosome-level scaffolds, confirmed by Hi-C data, are named according to size (  Figure 2;  Table 3).

**Table 3.** Chromosomal pseudomolecules in the primary genome assembly of *Rhopaloeides odorabile* odRhoOdor1.

INSDC accession	Molecule	Length (Mb)	GC%
OZ174347.1	1	36	37
OZ174348.1	2	24.45	36.50
OZ174349.1	3	21.90	37.50
OZ174350.1	4	19.04	37
OZ174351.1	5	17.88	37.50
OZ174352.1	6	16.92	37
OZ174353.1	7	16.41	37.50
OZ174354.1	8	16.33	38
OZ174355.1	9	15.75	36.50
OZ174356.1	10	15.16	37
OZ174357.1	11	14.44	37
OZ174358.1	12	12.55	37.50
OZ174359.1	13	12.22	37
OZ174360.1	14	12.09	37
OZ174361.1	15	11.94	37.50
OZ174362.1	16	11.93	37
OZ174363.1	17	11.27	37

**Table 4.** Software versions and sources.

Software	Version	Source
**BEDTools**	2.30.0	https://github.com/arq5x/bedtools2
**bin3C**	0.3.3	https://github.com/cerebis/bin3C
**BLAST**	2.14.0	ftp://ftp.ncbi.nlm.nih.gov/blast/executables/blast+/
**BlobToolKit**	4.3.9	https://github.com/blobtoolkit/blobtoolkit
**BUSCO**	5.5.0	https://gitlab.com/ezlab/busco
**bwa-mem2**	2.2.1	https://github.com/bwa-mem2/bwa-mem2
**checkM**	2015-01-16	https://ecogenomics.github.io/CheckM/
**Cooler**	0.8.11	https://github.com/open2c/cooler
**DIAMOND**	2.1.8	https://github.com/bbuchfink/diamond
**dRep**	3.4.0	https://github.com/MrOlm/drep
**fasta_windows**	0.2.4	https://github.com/tolkit/fasta_windows
**FastK**	1.1	https://github.com/thegenemyers/FASTK
**Gfastats**	1.3.6	https://github.com/vgl-hub/gfastats
**GenomeScope2.0**	2.0.1	https://github.com/tbenavi1/genomescope2.0
**GTDB-Tk **	1.2.1	https://github.com/Ecogenomics/GTDBTk
**Hifiasm**	0.19.8-r603	https://github.com/chhylp123/hifiasm
**HiGlass**	1.13.4	https://github.com/higlass/higlass
**MAGScoT**	1.0.0	https://github.com/ikmb/MAGScoT
**MaxBin**	2.2.7	https://sourceforge.net/projects/maxbin/
**MerquryFK**	1.1.2	https://github.com/thegenemyers/MERQURY.FK
**MetaBAT2**	2.15-15-gd6ea400	https://bitbucket.org/berkeleylab/metabat
**metaMDBG**	Pre-release	https://github.com/GaetanBenoitDev/metaMDBG
**metaTOR**	Pre-release	https://github.com/koszullab/metaTOR
**Minimap2**	2.24-r1122	https://github.com/lh3/minimap2
**MitoHiFi**	2	https://github.com/marcelauliano/MitoHiFi
**MultiQC**	1.14; 1.17 and 1.18	https://github.com/MultiQC/MultiQC
**Nextflow**	23.10.0	https://github.com/nextflow-io/nextflow
**PretextSnapshot**	0.0.5	https://github.com/sanger-tol/PretextSnapshot
**PretextView**	1.0.3	https://github.com/sanger-tol/PretextView
**Prokka**	1.14.5	https://github.com/tseemann/prokka
**Seqtk**	1.3	https://github.com/lh3/seqtk
**Singularity**	3.9.0	https://github.com/sylabs/singularity
**sanger-tol/ascc**	0.1.0	https://github.com/sanger-tol/ascc
**sanger-tol/blobtoolkit**	0.6.0	https://github.com/sanger-tol/blobtoolkit
**sanger-tol/curationpretext**	1.4.2	https://github.com/sanger-tol/curationpretext
**TreeVal**	1.4.0	https://github.com/sanger-tol/treeval
**YaHS**	1.1a.2	https://github.com/c-zhou/yahs

The mitochondrial genome was also assembled (length 16.42 kb, OZ174364.1). This sequence is included as a contig in the multifasta file of the genome submission and as a standalone record.

### Assembly quality metrics

**Figure 3.**
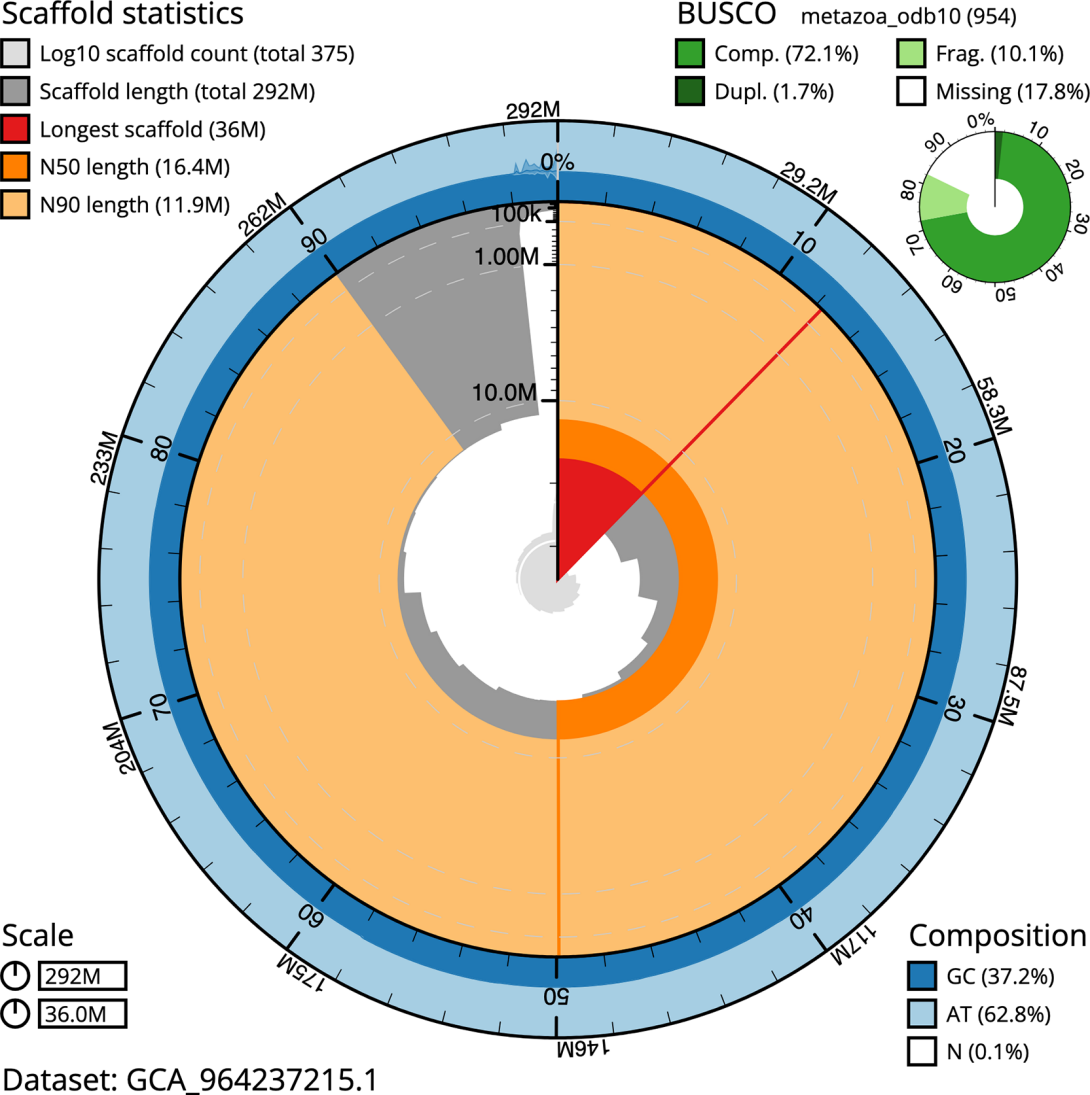
Assembly metrics for odRhoOdor1.1. The BlobToolKit snail plot provides an overview of assembly metrics and BUSCO gene completeness. The circumference represents the length of the whole genome sequence, and the main plot is divided into 1 000 bins around the circumference. The outermost blue tracks display the distribution of GC, AT, and N percentages across the bins. Scaffolds are arranged clockwise from longest to shortest and are depicted in dark grey. The longest scaffold is indicated by the red arc, and the deeper orange and pale orange arcs represent the N50 and N90 lengths. A light grey spiral at the centre shows the cumulative scaffold count on a logarithmic scale. A summary of complete, fragmented, duplicated, and missing BUSCO genes in the metazoa_odb10 set is presented at the top right. An interactive version of this figure can be accessed on the BlobToolKit viewer.

**Figure 4.**
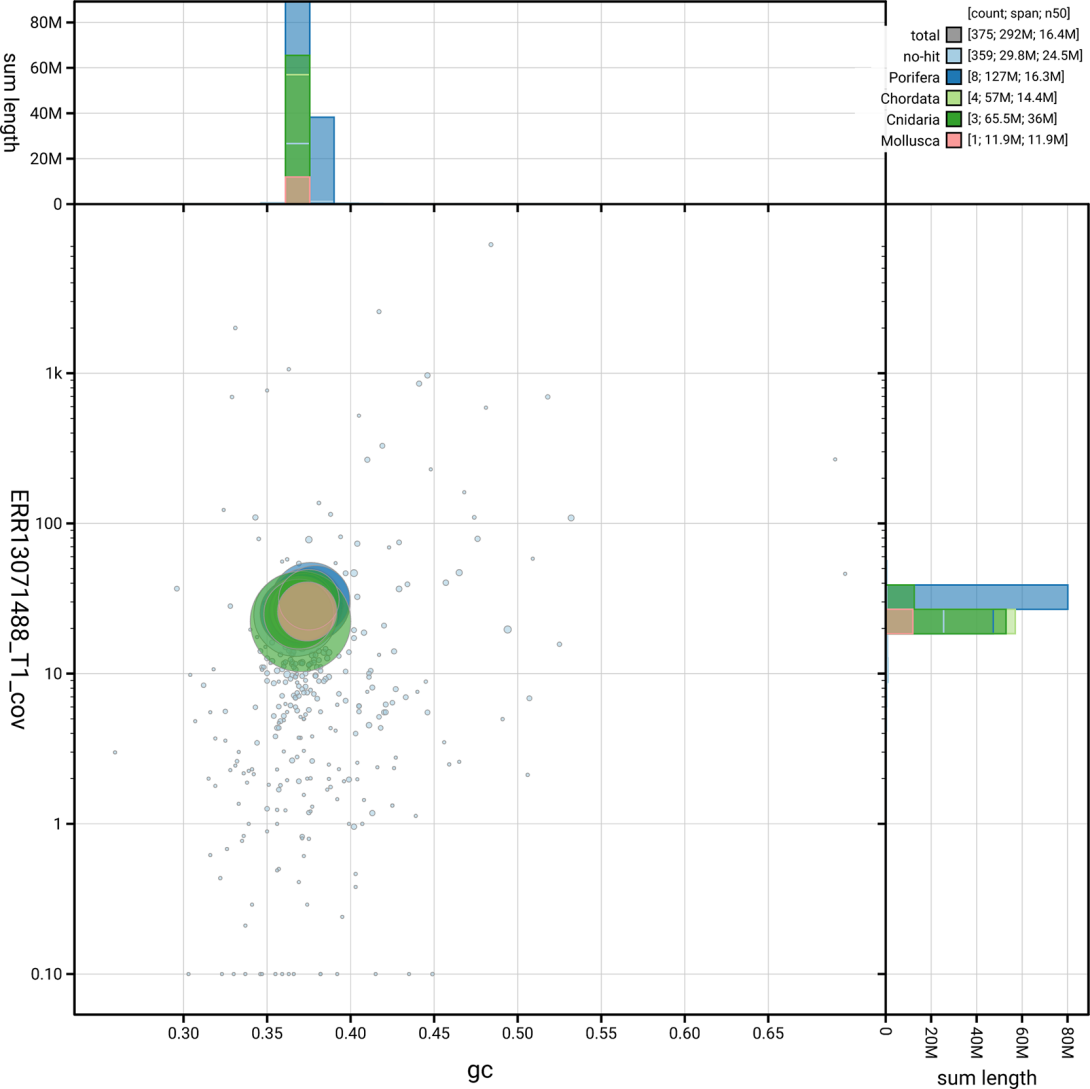
BlobToolKit blob plot for odRhoOdor1.1. The plot shows base coverage (vertical axis) and GC content (horizontal axis). The circles represent scaffolds, with the size proportional to scaffold length and the colour representing phylum membership. The histograms along the axes display the total length of sequences distributed across different levels of coverage and GC content. An interactive version of this figure is available on the BlobToolKit viewer.

The snail plot in  Figure 3 summarises the scaffold length distribution and other assembly statistics for the primary assembly. The blob plot in  Figure 4 shows the distribution of scaffolds by GC proportion and coverage.

BUSCO v.5.5.0 analysis using the metazoa_odb10 reference set ( *n* = 954) identified 72.1% of the expected gene set (single = 70.4%, duplicated = 1.7%).

### Taxonomic verification

We compared the C-Region of the 28S rDNA, currency regarded as most suitable barcoding marker for Demospongiae (see Voigt & Wörheide, 2016) against other samples identified as *Rhopaloeides odorabile* for this marker (however no type material), and other Dictyoceratida ( Erpenbeck *et al.*, 2020). Phylogenetic reconstruction recovers the sequence of this sample inside a clade of monophyletic *Rhopaloeides odorabile*, supporting its current taxonomic placement.

## Metagenome report

**Figure 5.**
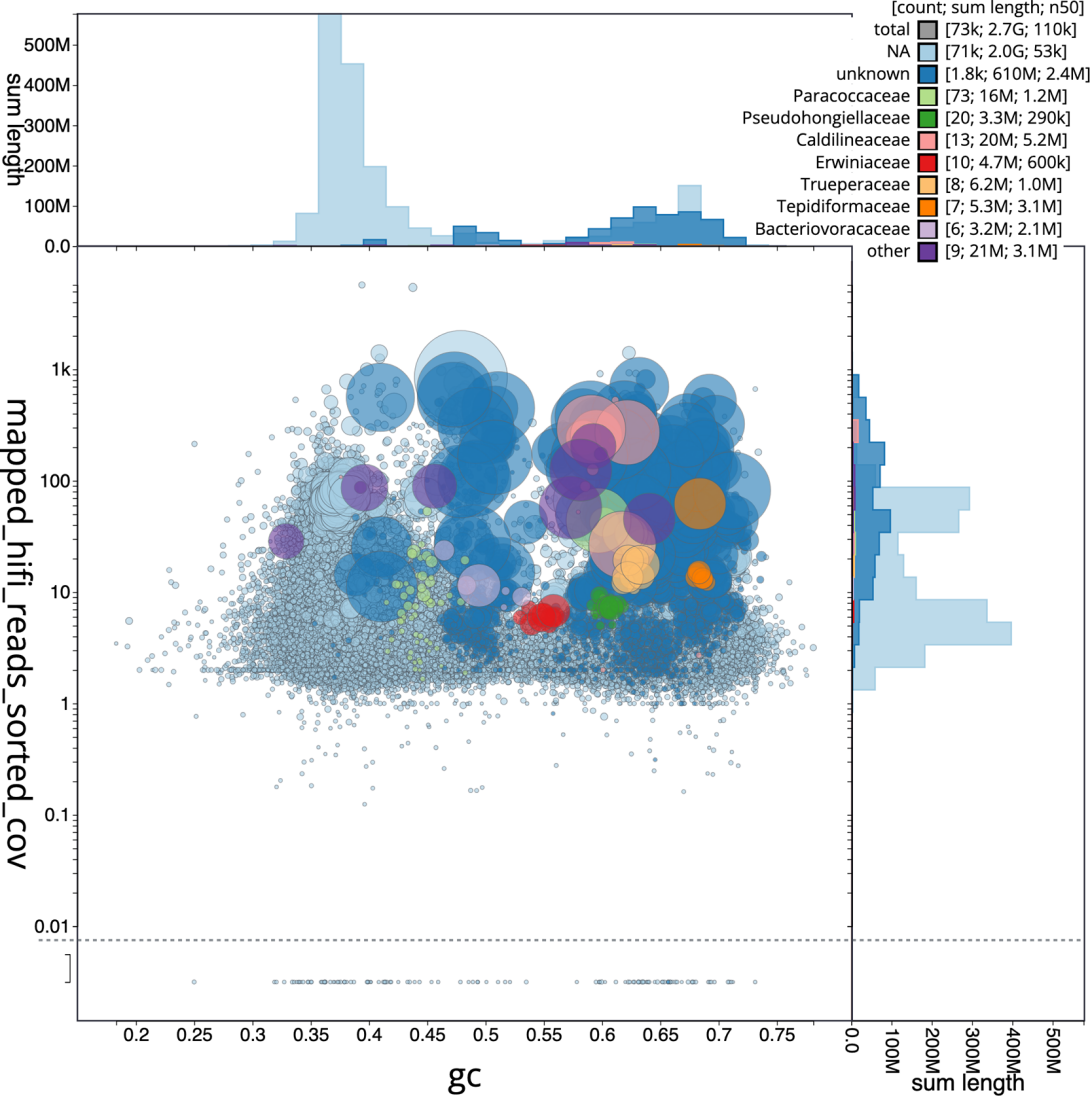
Blob plot for sequences in the *Rhopaloeides odorabile* metagenome. The plot shows base coverage (vertical axis) and GC content (horizontal axis). Binned contigs are coloured by family. Circles are sized in proportion to sequence length on a square-root scale, ranging from 510 to 6 879 066. Histograms show the distribution of sequence length sum along each axis. An interactive version of this figure may be viewed here.

We recovered 162 bins from the metagenome assembly ( Figure 5), of which 96 met the criteria for MAGs, including 27 fully circularised genomes. The recovered bins represented 19 bacterial and archaeal phyla, with genome sizes ranging from 0.85 to 8.40 Mbp (mean: 4.26 ± 1.42 Mbp). Mean completeness was 89.1% (± 11.2%) with 2.2% (± 2.2%) contamination.

**Figure 6.**
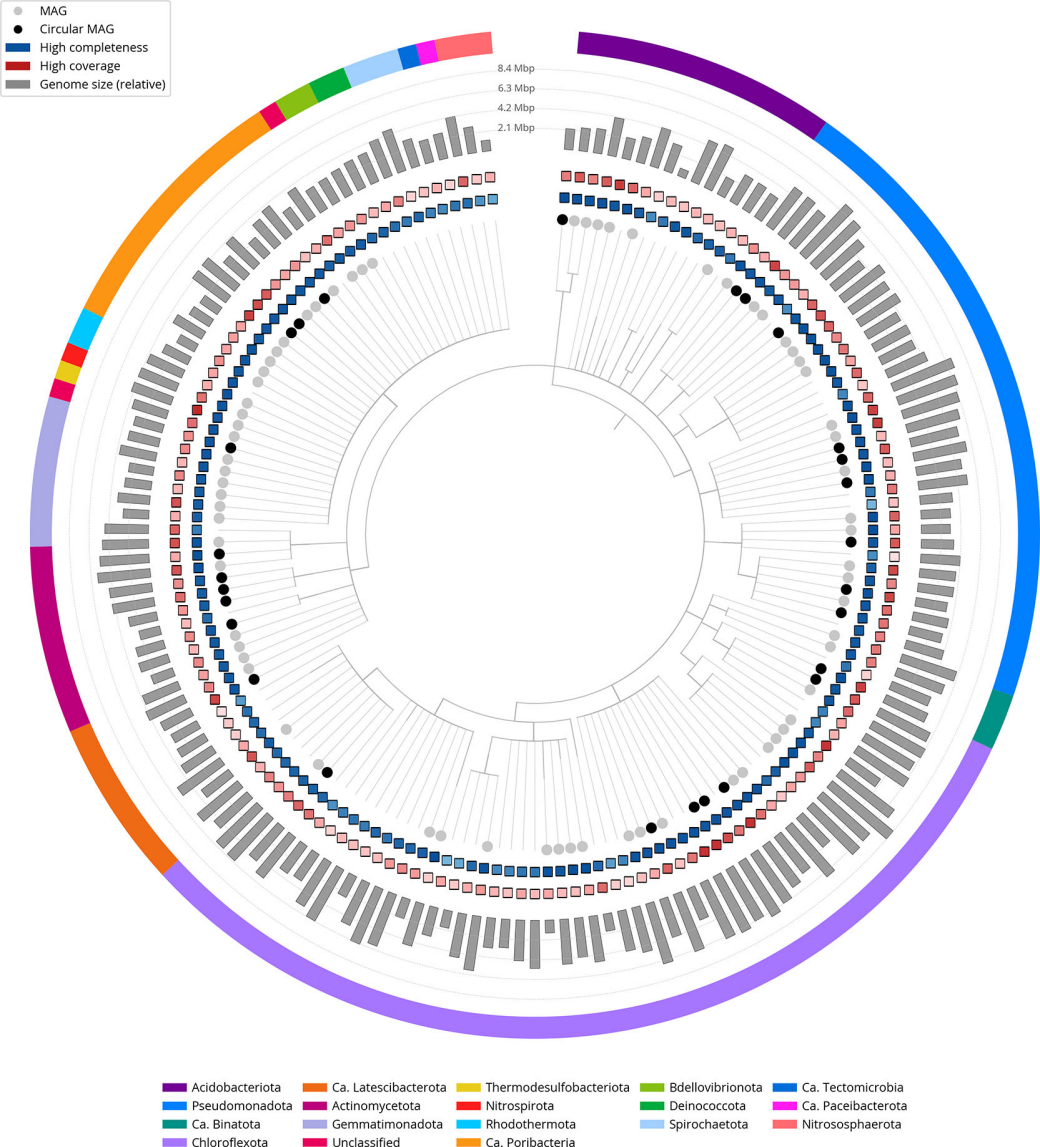
Taxonomic tree based on taxonomic classifications of metagenome bins, constructed using ete3. Colours indicate phylum-level taxonomy. Tracks show genome completeness (blue), sequencing coverage (red, log _10_), and genome size (grey bars, Mbp). High-quality MAGs are marked with grey circles; fully circularised MAGs in black.

Figure 6 summarises the taxa and quality of the metagenome bins. The full per-bin table of taxa and quality metrics for the metagenome bins is available on Zenodo.

## Author information

Contributors are listed at the following links:

• Members of the Wellcome Sanger Institute Tree of Life Management, Samples and Laboratory team
• Members of Wellcome Sanger Institute Scientific Operations – Sequencing Operations
• Members of the Wellcome Sanger Institute Tree of Life Core Informatics team
• Members of the EBI Aquatic Symbiosis Genomics Data Portal Team
• The Aquatic Symbiosis Genomics Project leadership

## Wellcome Sanger Institute – Legal and governance

The materials that have contributed to this genome note have been supplied by a Tree of Life collaborator. The Wellcome Sanger Institute employs a process whereby due diligence is carried out proportionate to the nature of the materials themselves, and the circumstances under which they have been/are to be collected and provided for use. The purpose of this is to address and mitigate any potential legal and/or ethical implications of receipt and use of the materials as part of the research project, and to ensure that in doing so we align with best practice wherever possible.

The overarching areas of consideration are:

• Ethical review of provenance and sourcing of the material
• Legality of collection, transfer and use (national and international)

Each transfer of samples is undertaken according to a Research Collaboration Agreement or Material Transfer Agreement entered into by the Tree of Life collaborator, Genome Research Limited (operating as the Wellcome Sanger Institute) and in some circumstances other Tree of Life collaborators.

## Data Availability

European Nucleotide Archive: Rhopaloeides odorabile. Accession number PRJEB75563. The genome sequence is released openly for reuse. The *Rhopaloeides odorabile* genome sequencing initiative is part of the Aquatic Symbiosis Genomics Project (PRJEB43743) and Sanger Institute Tree of Life Programme (PRJEB43745). All raw sequence data and the assembly have been deposited in INSDC databases. The genome will be annotated using available RNA-Seq data and presented through the Ensembl pipeline at the European Bioinformatics Institute. Raw data and assembly accession identifiers are reported in  Tables 1 and 2. Production code used in genome assembly at the WSI Tree of Life is available at https://github.com/sanger-tol . Results of provisional genome annotation are available on the ASG github repository. Table 4 lists software versions used in this study.
