# Supplementary material for: The chromosomal genome sequence of the sponge, Rhopaloeides odorabile Thompson, Murphy, Bergquist & Evans, 1987 (Dictyoceratida: Spongiidae) and its associated microbial metagenome sequences
Source: Wellcome Open Res. 2026 Apr 10;11:211. [Version 1] doi: 10.12688/wellcomeopenres.26110.1 (PMC13224822; doi:10.12688/wellcomeopenres.26110.1)
Supplement: Peer review history

# Reviewer report: Reviewer response for version 1

**DOI:** 10.21956/wellcomeopenres.28752.r153344

**Published:** 2026-07-01

**Authors:** Jayan Duminda M Senevirathna

**Affiliations:** 1 Uva Wellassa University, Badulla, Uva Province, Sri Lanka

The manuscript is well written, scientifically sound, and successfully addresses the objectives of the study. The methodology is appropriate, the results are clearly presented, and the conclusions are well supported by the data. I did not identify any major scientific or technical issues that require revision.

 I recommend acceptance in its current form, as the manuscript is suitable for indexing without further revision.

Are sufficient details of methods and materials provided to allow replication by others?

Yes

Is the rationale for creating the dataset(s) clearly described?

Yes

Are the datasets clearly presented in a useable and accessible format?

Yes

Are the protocols appropriate and is the work technically sound?

Yes

Reviewer Expertise:

Aquatic molecular biology and biotechnology

I confirm that I have read this submission and believe that I have an appropriate level of expertise to confirm that it is of an acceptable scientific standard.

# Reviewer report: Reviewer response for version 1

**DOI:** 10.21956/wellcomeopenres.28752.r153343

**Published:** 2026-05-11

**Authors:** Nabil Amor

**Affiliations:** 1 University Tunis EL Manar, Tunis, Tunisia

The manuscript “The chromosomal genome sequence of the sponge, *Rhopaloeides odorabile *Thompson, Murphy, Bergquist & Evans, 1987 (Dictyoceratida: Spongiidae) and its associated microbial metagenome sequences” presents a chromosome-level genome asseembly and associated microbial metagenome of yje marine sponge *Rhopaloeides odorabile. *This study provides valuable geneomic data for sponge biology, symbiosis andmarine ecologie. The manuscript is well structured, technically robust, and highly relevant for the genomics and symbiosis research community.

 Below are detailed comments and suggestions:

 Demospongiiae ◻ Demospongiae

 Why the reported BUSCO completeness is low? Is it due to genuine divergence of sponge genes? Assembly fragmentation?..

 The manuscript provides assembly stats but almost no biological interpretation of the genome. The manuscript would benifit from predicted gene number, proportion of the coding regions…

 The metagenome analysis should be added in the objectif section

 “We compared the C-Region of the 28S rDNA, currency regarded as most suitable barcoding marker “  do you mean currently

 How the overall assembly quality improved despite the lower N50?

 The manuscipt is a highly valuable genomic reference for sponge biology and marine symbiosis research

Are sufficient details of methods and materials provided to allow replication by others?

Yes

Is the rationale for creating the dataset(s) clearly described?

Yes

Are the datasets clearly presented in a useable and accessible format?

Yes

Are the protocols appropriate and is the work technically sound?

Yes

Reviewer Expertise:

Bioinformatics, OMICS, Phylogeny

I confirm that I have read this submission and believe that I have an appropriate level of expertise to confirm that it is of an acceptable scientific standard.

# Reviewer report: Reviewer response for version 1

**DOI:** 10.21956/wellcomeopenres.28752.r154202

**Published:** 2026-05-09

**Authors:** Soo-Je Park

**Affiliations:** 1 Jeju National University, Jeju-si, Jeju-do, South Korea

In this study, the authors described the genomic features of Rhopaloeides odorabile collected from Davies Reef and the MAGs reconstructed from its holobiont. The manuscript is generally well written, and the RNA-seq dataset will likely be a valuable resource for future studies. Overall, the publication of this data note is well justified.

 1. Please clarify the purpose of the RNA sequencing (simply) described in the Methods section. It is currently unclear whether the RNA-seq data were primarily used for genome annotation, transcript validation, or other analyses. Nonetheless, these data will provide a useful resource for future genomic and transcriptomic studies.

 2. Table 2 describes the assembly level as “chromosome.” Please clarify whether this corresponds to a “complete” or “chromosome-level” assembly according to INSDC/NCBI standards. In addition, the manuscript frequently uses the term “chromosomal pseudomolecules,” which may cause confusion for some readers. A brief explanation or clarification of this terminology would improve readability.

 3. Figure 6 would benefit from improved visualization. Consider providing a heatmap-style representation for completeness and coverage values. In addition, it is difficult to distinguish non-labelled tips in the phylogenomic tree except for MAGs and circular MAGs. Please improve figure clarity and labeling.

 Typo:

 P3, Demospongiiae à Demospongiae ?

 P12, as most suitable à as the most suitable ?

Are sufficient details of methods and materials provided to allow replication by others?

Yes

Is the rationale for creating the dataset(s) clearly described?

Yes

Are the datasets clearly presented in a useable and accessible format?

Yes

Are the protocols appropriate and is the work technically sound?

Yes

Reviewer Expertise:

Metagenomics, Ecogenomics, Environmental microbiology

I confirm that I have read this submission and believe that I have an appropriate level of expertise to confirm that it is of an acceptable scientific standard.
